# Development and validation of a blood biomarker risk prediction model for postmenopausal endometrioid endometrial cancer

**DOI:** 10.3389/fendo.2026.1820474

**Published:** 2026-07-03

**Authors:** Hubin Xu, Mengyu Zhang, Huinan Zhu, Haimin Jiang, Wenjie Zeng, Xiaoyan Chen, Huafeng Shou, Lingqian Zhao

**Affiliations:** 1 The Second School of Clinical Medicine, Hangzhou Normal University, Hangzhou, Zhejiang, China; 2 Center for Reproductive Medicine, Department of Gynecology, Zhejiang Provincial People’s Hospital (Affiliated People’s Hospital, Hangzhou Medical College), Hangzhou, Zhejiang, China

**Keywords:** endometrial polyps, endometrioid endometrial carcinoma, nomogram, postmenopausal women, preoperative peripheral venous blood biomarkers

## Abstract

**Objective:**

To investigate the association between preoperative peripheral venous blood biomarkers and postmenopausal endometrioid endometrial carcinoma (EEC) and to develop a clinical risk prediction model.

**Methods:**

A retrospective study was conducted on patients who underwent hysteroscopic examination in the Department of Gynecology, Zhejiang Provincial People’s Hospital, between 2018 and 2024. Clinical data, pathological findings, and peripheral venous blood test results were collected. The t-test and least absolute shrinkage and selection operator (LASSO) regression analysis were performed to identify potential risk factors associated with postmenopausal EEC. Logistic regression analysis was subsequently conducted to determine independent risk factors. A clinical risk prediction model was developed based on these factors, and its discriminative and calibration abilities were assessed. Finally, the model was deployed in an online calculator for clinical application.

**Results:**

After applying inclusion and exclusion criteria, a total of 311 patients were enrolled, including 119 cases of benign endometrial diseases and 192 cases of endometrioid carcinoma. Through t-test analysis, LASSO regression, and logistic regression analysis, five independent risk factors were identified: body mass index (BMI), cancer antigen 125 (CA125), human epididymis protein 4 (HE4), platelet-to-lymphocyte ratio (PLR), and albumin (ALB). A clinical prediction model incorporating these factors was established, yielding an area under the curve (AUC) of 0.936 (95% CI: 0.9081–0.9631). At the optimal Youden index, the model demonstrated a specificity of 89.1% and a sensitivity of 90.1%, indicating excellent discriminative and calibration performance. The final model was implemented in an online calculator to facilitate dynamic clinical risk predictions for postmenopausal EEC.

**Conclusion:**

BMI, CA125, HE4, PLR, and ALB were identified as independent risk factors for postmenopausal EEC. The clinical risk prediction model based on preoperative blood biomarkers demonstrated robust predictive performance, supporting its potential application in clinical decision-making.

## Introduction

In recent years, the latest statistics from the American Cancer Society show that endometrial cancer (EC), the main subtype of uterine cancer, is the fourth most common malignant tumor in women and the fifth leading cause of cancer-related death. Over the past 40 years, the overall survival rate of EC has not improved significantly, while the mortality rate has continued to rise (1). The histological type of EC is mainly endometrioid endometrial carcinoma (EEC), accounting for about 75% of all EC cases (2). In addition, epidemiological studies have shown that postmenopausal women account for more than 80% of EC patients (3). Early identification of high-risk individuals and timely pathological diagnosis are important for improving the prognosis of postmenopausal women with EEC. However, currently used diagnostic modalities, including transvaginal ultrasound (TVS), magnetic resonance imaging (MRI), and hysteroscopic biopsy, are associated with certain limitations. These include limited specificity for early lesions, procedural invasiveness in the case of hysteroscopy, and high cost, which collectively restrict their routine use in broad clinical risk assessment settings.

In recent years, research on tumor diagnosis and prognosis prediction based on peripheral blood markers has made significant progress. Among them, inflammation-related indicators have received widespread attention due to their key role in the occurrence and development of cancer (4, 5). Chronic inflammation can drive the occurrence and progression of tumors by promoting the release of a variety of tumor-promoting factors (6, 7). Typical inflammatory immune cells, such as neutrophils, lymphocytes, and the lymphocyte-to-monocyte ratio (LMR) and platelet-to-lymphocyte ratio (PLR) calculated based on their ratios, have been shown to have good predictive value in predictive models for various cancers such as lung cancer, colorectal cancer, and urinary system malignancies (8–11). However, the current research on the predictive value of peripheral blood biomarkers in EEC is still limited and lacks the support of long-term clinical data. Therefore, this study aimed to investigate the association between peripheral blood inflammatory markers and benign and malignant endometrial lesions in postmenopausal women, and to develop a clinical risk prediction model for postmenopausal EEC.

## Materials and methods

This study retrospectively collected clinical and pathological data of postmenopausal female patients who visited the Department of Gynecology of Zhejiang Provincial People’s Hospital and underwent hysteroscopy from October 2018 to October 2024.

Inclusion criteria: postmenopausal women; underwent hysteroscopy and obtained endometrial tissue for pathological evaluation.

Exclusion criteria:① Previous or concurrent malignant tumors, or a history of pelvic and abdominal radiotherapy; ② Accompanied by hematologic disorders, such as aplastic anemia, megaloblastic anemia, lymphoma, etc.; ③ Accompanied by autoimmune diseases, such as systemic lupus erythematosus, immune thrombocytopenic purpura, etc.; ④ Preoperative acute or chronic inflammation, or other diseases that affect routine blood tests, such as acute or chronic infections of the upper respiratory tract, digestive tract, urinary tract, etc.; ⑤ Incomplete preoperative clinical data, peripheral blood test data, or pathological data, such as incomplete data due to incomplete test or examination items or different recording methods.

The general clinical information (age, height, weight) of all enrolled patients was collected, and the body mass index (BMI) was calculated. Peripheral venous blood samples were collected from patients in the morning in the fasting state one week before surgery for blood cell analysis. The laboratory indicators collected manually include: tumor markers: neuron-specific enolase (NSE), alpha-fetoprotein (AFP), carcinoembryonic antigen (CEA), carbohydrate antigen 125 (CA125), carbohydrate antigen 199 (CA199), carbohydrate antigen 153 (CA153), squamous cell carcinoma antigen (SCC), cytokeratin 19 (CK19), human epididymis protein 4 (HE4), pro-gastrin-releasing peptide (ProGRP); hematological indicators: lymphocyte count, monocyte count, neutrophil count, platelet count; biochemical indicators: serum albumin (ALB), fasting blood glucose (FBG), total cholesterol (total cholesterol, TC), triglyceride (TG), high-density lipoprotein (HDL), low-density lipoprotein (LDL), and non-high-density lipoprotein (Non-HDL). In addition, the following inflammation and nutrition-related indices were calculated and recorded: NLR (neutrophil-to-lymphocyte ratio); PLR (platelet-to-lymphocyte ratio); LMR (lymphocyte-to-monocyte ratio); SII (systemic immune-inflammation index, calculated by the formula: platelet count × neutrophil count/lymphocyte count); PNI (prognostic nutritional index, calculated by the formula: ALB + 5 × lymphocyte count); GNRI (modified nutritional risk index, calculated by the formula: 1.489 × ALB + 41.7 × body weight/(21 × height²)); TyG index (triglyceride-glucose index, calculated by the formula: ln(TG × FBG/2)). The measurement data were expressed as mean ± standard deviation ( $\bar{x}$ ± S), and the count data were expressed as frequency and percentage.

All data were statistically analyzed using SPSS 26.0, R Studio 1.4 and related extension packages. Independent sample t-test was used to compare the two groups of quantitative data, and risk factors related to postmenopausal endometrioid carcinoma in preoperative blood markers and clinical information were preliminarily screened. LASSO (least absolute shrinkage and selection operator) regression analysis was used to further screen key variables. Cross-validation was used to determine the optimal penalty parameter (λ) of LASSO regression, and the maximum λ value (lambda.1se) within the range of one standard deviation of the mean error was selected, and finally the variables with non-zero regression coefficients were screened. The risk factors screened by LASSO regression were included in the logistic regression analysis to further determine the independent risk factors of postmenopausal EEC, and the odds ratio (OR) and its 95% confidence interval (95% CI) were calculated.

Logistic regression was used to construct a clinical prediction model based on independent risk factors. The predictive performance of the model was evaluated by the receiver operating characteristic (ROC) curve, and the area under the curve (AUC), 95% CI, Youden index, sensitivity, specificity, positive predictive value, negative predictive value and accuracy were calculated. The Bootstrap method was used for 1000 internal validations, and the fit of the prediction model was evaluated by the calibration curve. The closer the correlation between the model prediction value and the actual value was to the 45° diagonal, the better the calibration ability of the prediction model. The clinical net benefit of the model was calculated by decision curve analysis (DCA), and the performance of the model curve, full intervention strategy (oblique arc) and no intervention strategy (horizontal line) under different threshold ranges was compared to evaluate the clinical applicability of the model.

Based on the established and verified clinical prediction model, an online calculator (web-based calculator) was developed using R language to achieve individualized risk prediction of postmenopausal EEC.

All statistical tests were two-sided tests, and P < 0.05 was statistically significant.

## Results

**Figure 1:**
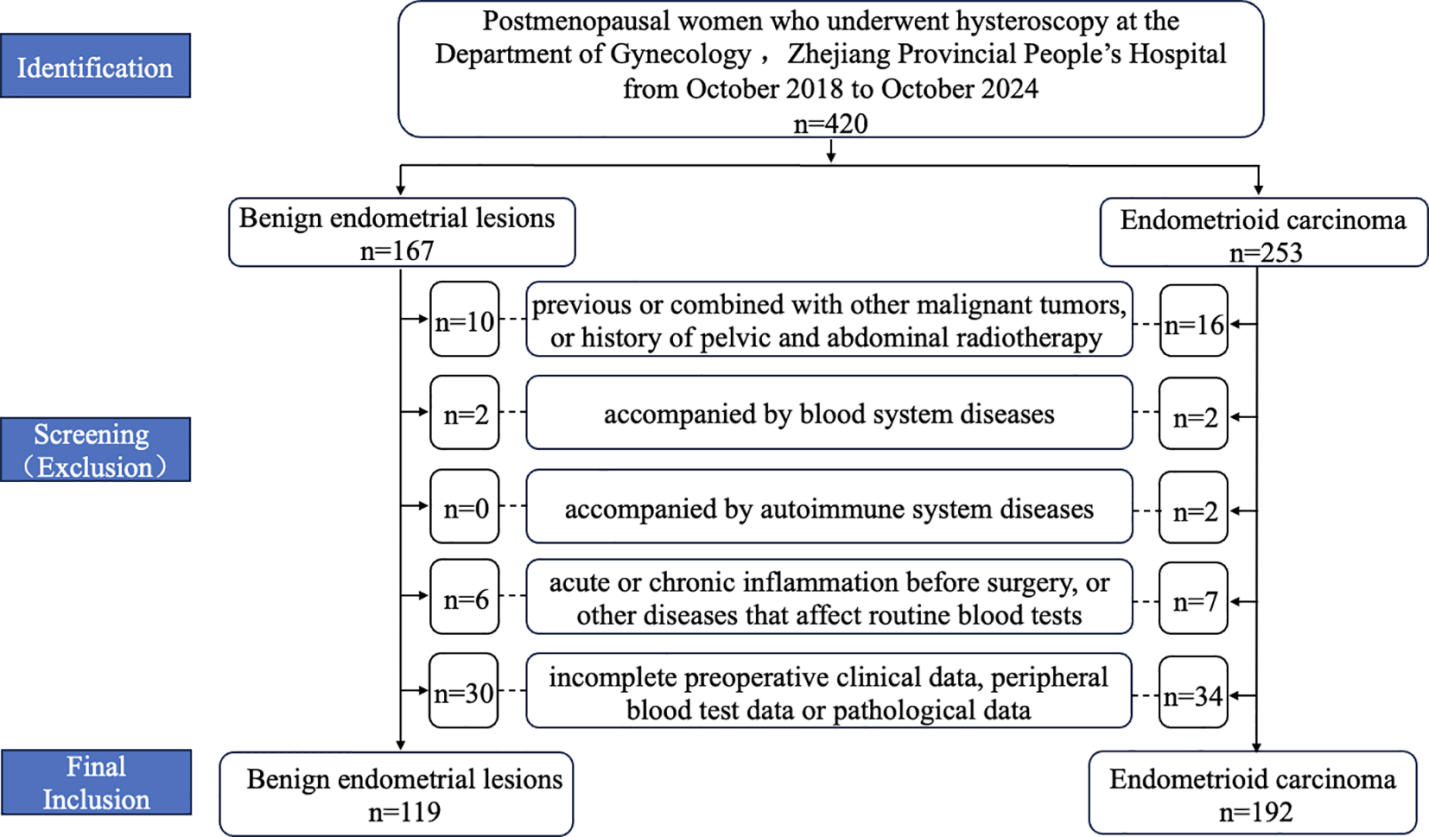
The inclusion and exclusion process.

According to the inclusion and exclusion criteria, a total of 311 postmenopausal women who underwent hysteroscopy were included in this study, including 119 cases (38.3%, including endometrial polyps (n=87), simple endometrial hyperplasia (n=19), endometrial atrophy (n=13)) with benign endometrial lesions and 192 cases (61.7%) with endometrioid carcinoma (EEC). The inclusion and exclusion process is detailed in **Figure 1**. The general clinical information of the patients and the levels of peripheral venous blood markers are shown in **Table 1**.

**Table 1:** General clinical information and peripheral venous blood marker levels in postmenopausal patients undergoing hysteroscopy.

	Reference interval	Total	Benign endometrial diseases	Endometrioid carcinoma
n(%)		311	119(38.3%)	192(61.7%)
Age		59.64 ± 7.93	58.91 ± 6.93	60.10 ± 8.48
Height (cm)		157.73 ± 4.54	158.03 ± 2.91	157.54 ± 5.31
Weight (kg)		62.22 ± 10.25	59.40 ± 8.92	63.97 ± 10.65
BMI (kg/m^2^)		24.98 ± 3.77	23.77 ± 3.34	25.74 ± 3.83
NSE (ng/mL)	≤20.0	12.71 ± 4.85	12.71 ± 1.57	12.71 ± 6.06
AFP (ng/mL)	≤20.0	3.28 ± 4.08	3.16 ± 1.02	3.36 ± 5.14
CEA (ng/mL)	≤1.70	2.48 ± 4.97	2.22 ± 1.16	2.65 ± 6.26
CA125 (U/mL)	≤35.0	25.04 ± 30.68	12.69 ± 6.37	32.69 ± 36.73
CA199 (U/mL)	≤37.0	34.23 ± 193.91	9.83 ± 6.10	49.36 ± 245.77
CA153 (U/mL)	≤31.3	12.72 ± 7.50	12.34 ± 4.76	12.96 ± 8.79
SCC (ng/mL)	≤3.0	1.05 ± 0.39	1.07 ± 0.31	1.04 ± 0.43
CK19 (ng/mL)	≤3.8	2.70 ± 1.65	2.19 ± 0.59	3.01 ± 1.98
CA724 (U/mL)	≤19.3	6.25 ± 21.65	4.91 ± 3.95	7.08 ± 27.37
HE4 (pmol/mL)	≤105.1	88.36 ± 84.03	48.67 ± 9.30	112.97 ± 99.08
ProGRP (pg/mL)	25.0-78.0	46.79 ± 15.88	46.50 ± 12.47	46.96 ± 17.70
LMR		5.97 ± 2.01	5.97 ± 1.91	5.97 ± 2.08
NLR		2.14 ± 1.19	2.09 ± 0.96	2.18 ± 1.31
PLR		137.33 ± 46.14	143.04 ± 48.98	133.79 ± 44.05
SII		516.82 ± 303.53	517.24 ± 280.56	516.56 ± 317.64
ALB (g/L)	65.0-85.0	41.63 ± 3.61	43.57 ± 1.94	40.44 ± 3.88
FBG (mmol/L)	3.92-6.16	5.63 ± 1.70	5.60 ± 1.10	5.65 ± 1.98
TC (mmol/L)	3.11-5.96	5.13 ± 0.93	5.19 ± 0.71	5.09 ± 1.04
TG (mmol/L)	0.34-1.70	1.74 ± 1.03	1.65 ± 0.75	1.80 ± 1.16
HDL (mmol/L)	1.10-1.74	1.23 ± 0.27	1.31 ± 0.25	1.18 ± 0.27
LDL (mmol/L)	<3.12	3.00 ± 0.71	2.99 ± 0.54	3.01 ± 0.80
Non-HDL (mmol/L)	0.86-4.10	3.88 ± 0.87	3.87 ± 0.67	3.89 ± 0.98
PNI		51.08 ± 4.75	52.74 ± 3.31	50.05 ± 5.20
GNRI		370.77 ± 60.93	360.11 ± 50.84	377.38 ± 65.68
Tyg		1.45 ± 0.55	1.44 ± 0.43	1.45 ± 0.62

The results of t-test analysis showed that preoperative weight, BMI, CA125, CA199, CK19, HE4, ALB, HDL, PNI and GNRI were significantly associated with postmenopausal EEC. In contrast, age, height, NSE, AFP, CEA, CA153, SCC, CA724, ProGRP, LMR, NLR, PLR, SII, FBG, TC, TG, LDL, Non-HDL and Tyg were not statistically significantly correlated with postmenopausal EEC (**Table 2**).

**Table 2:** T-test, LASSO regression analysis, and logistic regression analysis results.

	P (T-test)	Lasso.coef (LASSO regression analysis)	P (Logistic regression analysis)	OR (Logistic regression analysis)	95%CI (Logistic regression analysis)
Age	0.180				
Height (cm)	0.297				
Weight (kg)	<0.001				
BMI (kg/m^2^)	<0.001	0.079	0.009	1.146	1.035-1.269
NSE (ng/mL)	0.994				
AFP (ng/mL)	0.595				
CEA (ng/mL)	0.462				
CA125 (U/mL)	<0.001	0.034	0.001	1.089	1.038-1.143
CA199 (U/mL)	0.027	8.99E^-6^	0.611		
CA153 (U/mL)	0.421				
SCC (ng/mL)	0.442				
CK19 (ng/mL)	<0.001				
CA724 (U/mL)	0.281				
HE4 (pmol/mL)	<0.001	0.035	<0.001	1.070	1.043-1.097
ProGRP (pg/mL)	0.788				
LMR	0.991				
NLR	0.516				
PLR	0.086	-0.003	0.038	0.992	0.984-1.000
SII	0.985				
ALB (g/L)	<0.001	-0.219	<0.001	0.708	0.617-0.814
FBG (mmol/L)	0.769				
TC (mmol/L)	0.335				
TG (mmol/L)	0.178				
HDL (mmol/L)	<0.001	-0.524	0.309		
LDL (mmol/L)	0.822				
Non-HDL (mmol/L)	0.808				
PNI	<0.001				
GNRI	0.010				
Tyg	0.844				

**Figure 2:**
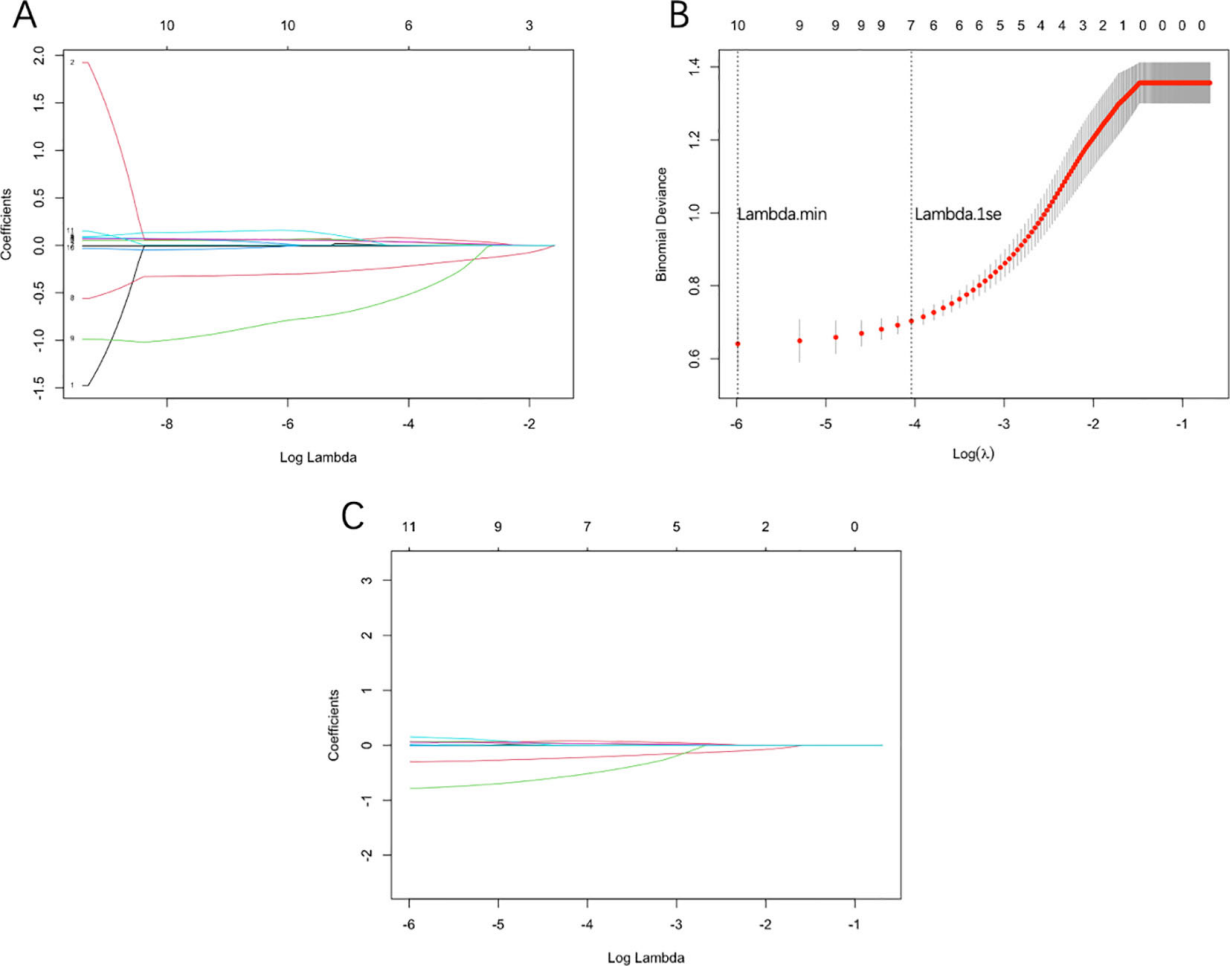
LASSO regression analysis to screen risk factors. [**(A)** LASSO regression coefficient path diagram; **(B)** LASSO regression cross-validation curve; **(C)** Variables screened by LASSO regression].

The significant risk factors of t-test are included in lasso regression. At the same time, PLR has been included in lasso regression because it has been proved to be relevant in most previous studies. The optimal penalty parameter (λ) was determined through 3-fold cross-validation. The minimum mean cross-validated error was achieved at λ = 0.000083. The coefficient trajectory against log(λ) is shown in **Figure 2A**, and the cross-validation curve is presented in **Figure 2B**. Two standard criteria for λ selection were evaluated, namely λ.min (minimum cross-validated error) and λ.1se (most regularized model within one standard error of the minimum error). At λ.1se, a total of seven variables retained non-zero coefficients, including BMI, CA125, CA199, HE4, PLR, ALB, and HDL.

**Figure 3:**
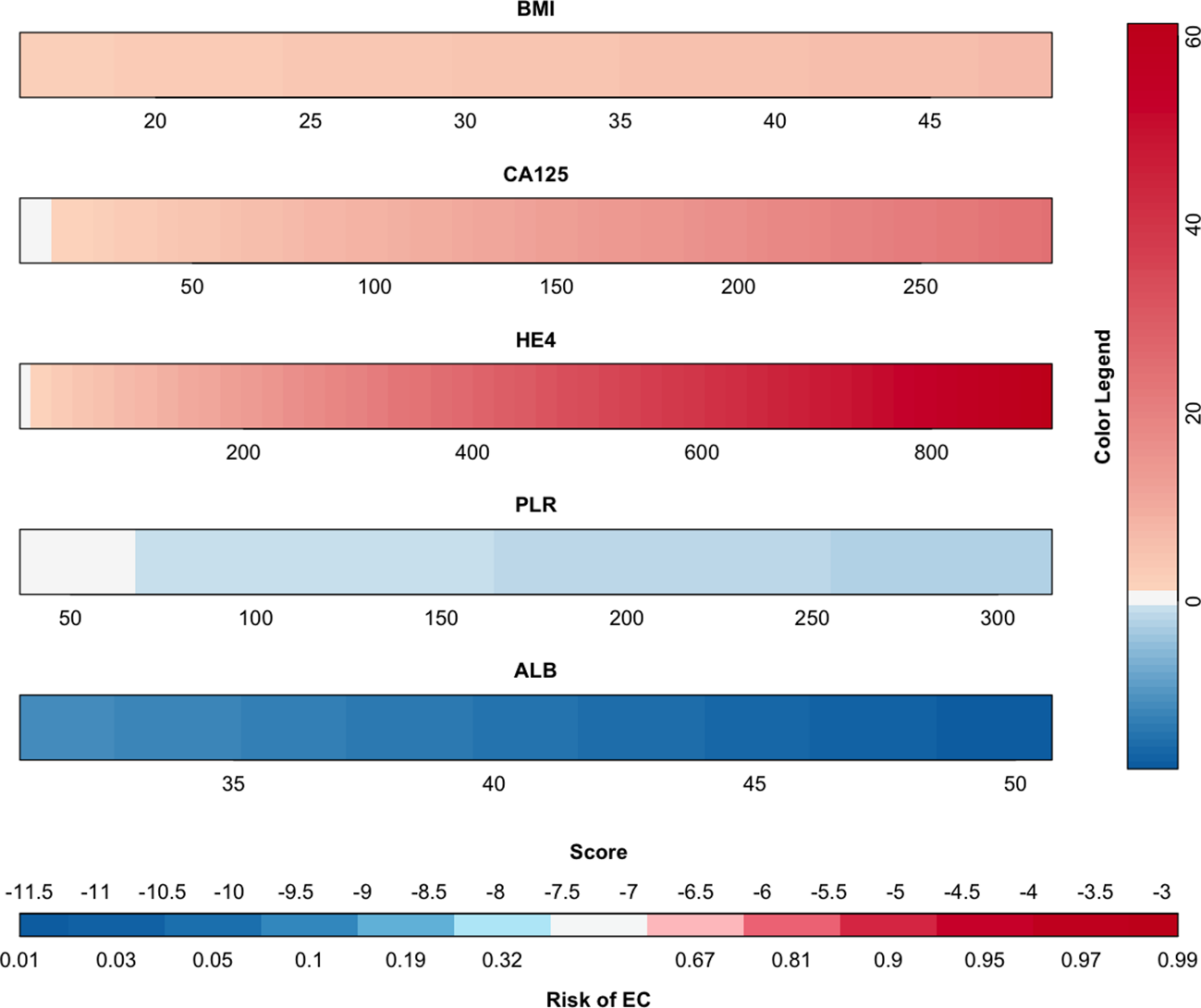
Clinical prediction model for postmenopausal EEC.

**Figure 4:**
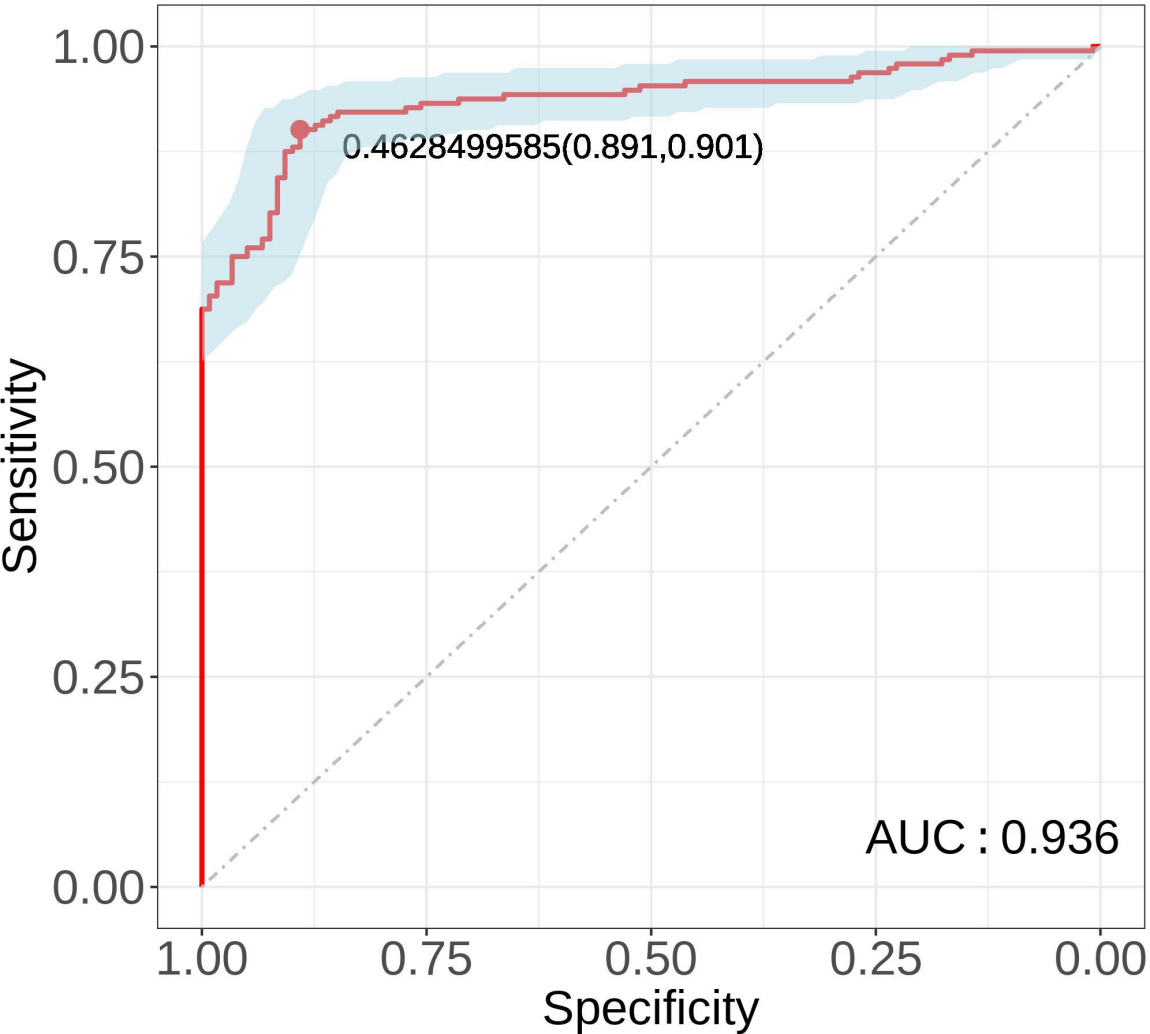
AUC curve of the clinical prediction model for postmenopausal EEC.

The risk factors identified through t-test and LASSO regression analysis comprised BMI, CA125, CA199, HE4, PLR, ALB, and HDL. Logistic regression analysis was subsequently conducted on these risk factors to further ascertain the independent risk factors. The results indicated that for CA199 (P = 0.611) and HDL (P = 0.309), thus, the independent risk factors were ultimately determined as follows: BMI (P = 0.009, OR = 1.146, 95% CI: 1.035–1.269), CA125 (P = 0.001, OR = 1.089, 95% CI: 1.038–1.143), HE4 (P < 0.001, OR = 1.070, 95% CI: 1.043–1.097), PLR (P = 0.038, OR = 0.992, 95% CI: 0.984–1.000),ALB (P < 0.001, OR = 0.708, 95% CI: 0.617–0.814). The above five indicators were finally confirmed as independent risk factors for EEC. A logistic regression prediction model was constructed based on BMI, CA125, HE4, PLR, and ALB. The specific model is as follows: 7.210 + 0.136*BMI(kg/m2)+0.085*CA125(U/mL)+0.067*HE4(pmol/mL)-0.008*PLR-0.345*ALB(g/L) (**Figure 3**). The model’s ability to distinguish was evaluated by the receiver operating characteristic (ROC) curve (**Figure 4**). The results showed that AUC = 0.936 (95% CI: 0.9081–0.9631), indicating that the model has good predictive efficacy. When the Youden index was the largest, the model had a specificity of 89.1% and a sensitivity of 90.1%, indicating its high classification ability. The positive predictive value (PPV) = 93.4%, the negative predictive value (NPV) = 83.6%, and the overall accuracy = 89.4%.

**Figure 5:**
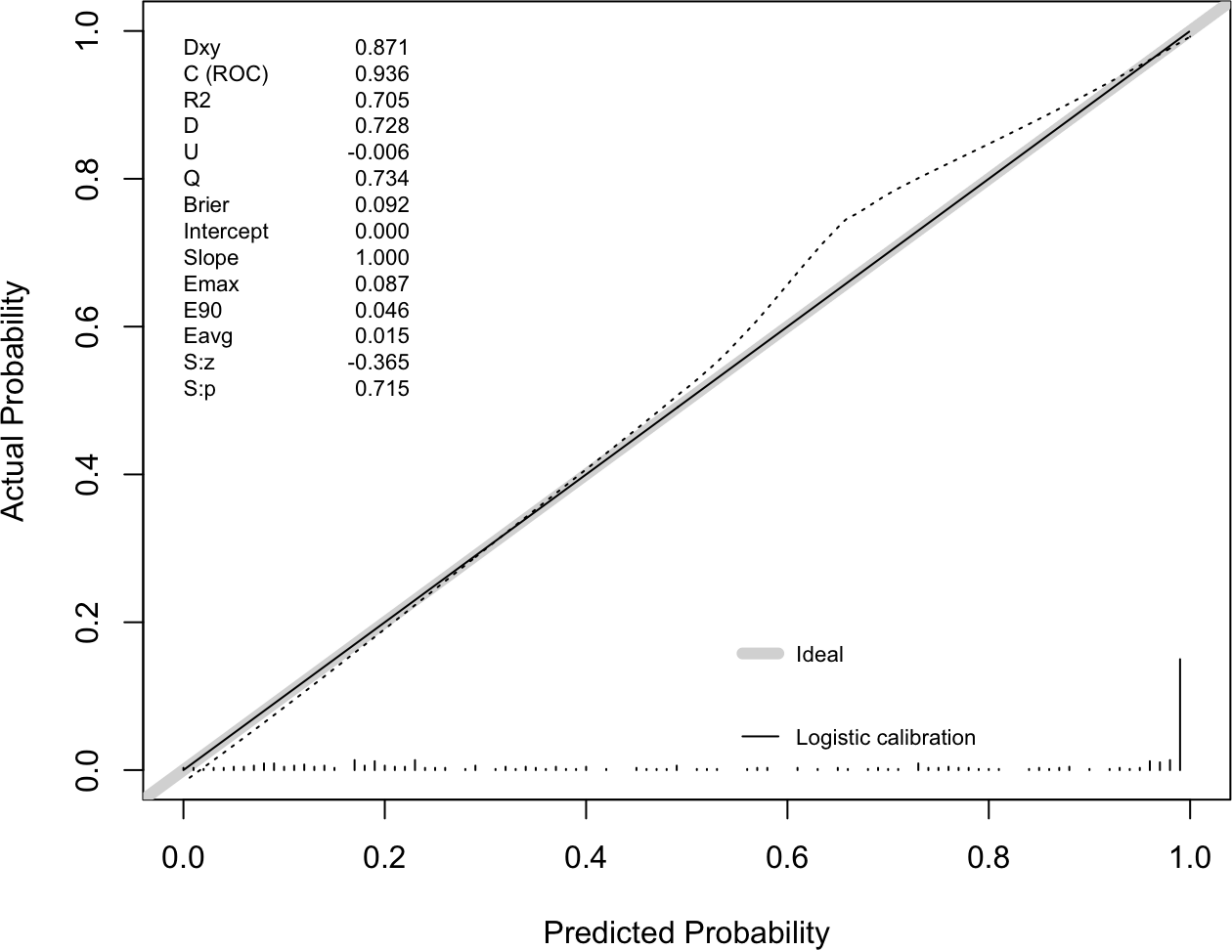
Calibration curve of clinical prediction model for postmenopausal EEC.

**Figure 6:**
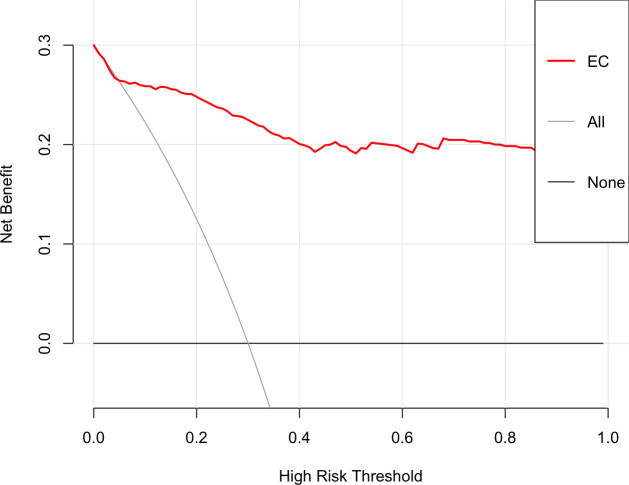
DCA curve of clinical prediction model for postmenopausal EEC.

In order to evaluate the reliability and clinical applicability of the model, internal validation and decision analysis were further performed. In the calibration curve (**Figure 5**), the unreliability index (U) = 0.006, indicating that the model has low unreliability, the quality index (Q) = 0.734, indicating that the model has high quality, Brier Score = 0.092, indicating accurate prediction, Emax = 0.087, E90 = 0.046, indicating that the model has a small error in calibration, all indicating that the model has high prediction accuracy and good calibration ability. Decision curve analysis (DCA) (**Figure 6**) showed that within the threshold probability range of 0–0.8, the intervention benefit of the model was significantly higher than that of the non-intervention or full intervention strategy, suggesting its potential application value in clinical decision-making.

**Figure 7:**
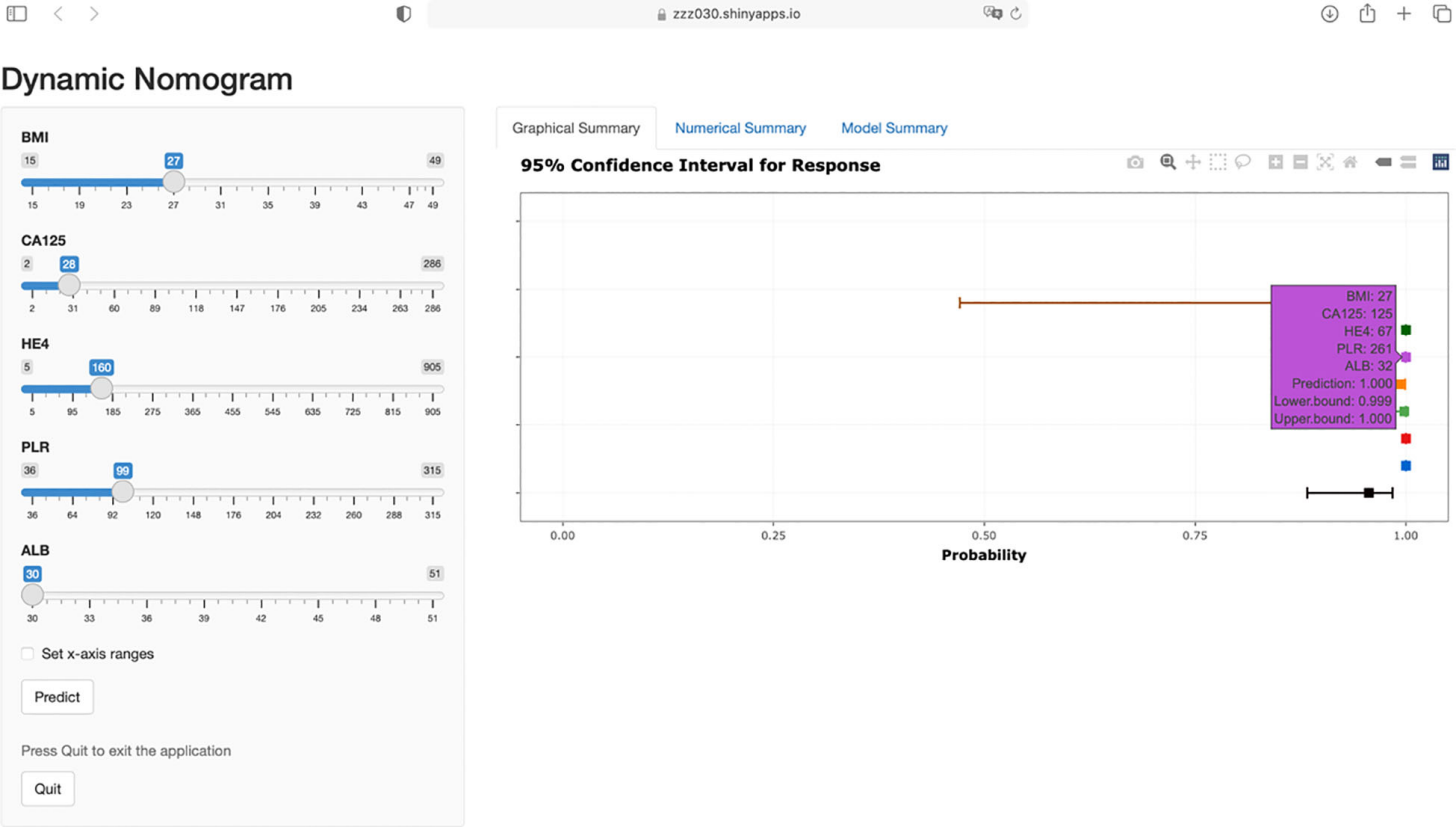
Dynamic model for clinical prediction of postmenopausal EEC - online calculator (https://zzz030.shinyapps.io/Postmenopausal-EndometrialCancer-DynNomapp/).

Finally, a dynamic prediction model for postmenopausal EEC was developed based on the R language environment and put into the online calculator (https://zzz030.shinyapps.io/Postmenopausal-EndometrialCancer-DynNomapp/). Users can input the patient’s clinical information to calculate the predicted risk of EEC. Through multiple sets of data simulation tests, the webpage runs stably, indicating that the online prediction tool has good performance (**Figure 7**).

## Discussion

Endometrioid endometrial carcinoma represents the most common histological subtype of endometrial cancer and predominantly occurs in postmenopausal women. Data show that the 5-year survival rate of patients with stage I EEC is more than 75% higher than that of patients with stage IV (12, 13), Its rising incidence worldwide and the marked survival disparity between early- and advanced-stage disease underscore the clinical importance of early identification in appropriately selected high-risk populations. Most of the early EEC had no clinical symptoms, and a few had symptoms such as increased secretion and abnormal bleeding after menopause, but the specificity was low. Transvaginal ultrasound (TVS) is the preferred screening tool, but its specificity for postmenopausal symptomatic patients is only 22%-62% (14). Hysteroscopic biopsy is considered to be the main method for the diagnosis of early endometrial cancer, but the incidence of surgical complications is as high as 3%-5%, and there is a certain risk of tumor spread (15). And the above methods are affected by the operator’s experience and subjective judgment, with low consistency (16). Molecular typing, as a molecular marker with high accuracy and recognition, affects the survival and prognosis of patients and participates in important clinical decisions. However, the cost of molecular detection is high, and its promotion in economically underdeveloped areas is limited.

Given these limitations, increasing attention has been paid to the development of accessible and minimally invasive biomarkers derived from peripheral blood and other biological specimens, including cervical scrapings (17, 18), vaginal secretions (19), urine (20), blood (21), etc. These methods have shown certain diagnostic value in preliminary studies, but due to limited data, their sensitivity and specificity, as well as their feasibility in clinical applications, need to be further verified.

In recent years, the role of inflammation and immunity in the tumor microenvironment has received extensive attention and has made significant contributions to the occurrence, progression, invasion, promotion and metastasis of tumors (6). Qin’s team (22) found in peripheral blood tests that NLR, PLR and MLR were significantly elevated in EC patients, and the diagnostic efficacy of combined detection was better than that of a single indicator. Marin et al. (23) also confirmed that the level of PLR was significantly correlated with the benign and malignant nature of endometrial hyperplasia through statistical analysis of 670 patients (192 with endometrial cancer and 478 with endometrial hyperplasia). In addition, Ronsini et al. (24) further proposed that the SIR-En index (calculated by the systemic inflammatory index SII × endometrial thickness) can effectively distinguish endometrial hyperplasia from cancer in women with postmenopausal abnormal uterine bleeding, and its ROC curve AUC was 0.6351 (95% CI: 0.5579-0.7121).

Obesity is a well-established risk factor for EC, particularly in postmenopausal women. Our findings further support BMI as an independent risk factor for EEC, consistent with extensive epidemiological evidence. The meta-analysis showed that for every 5 kg/m² increase in BMI, the relative risk of EC increased by 1.62 (99% CI: 1.56-1.69), and the higher the BMI, the more significant the association, especially in postmenopausal women (25). The association mechanism between obesity and EEC risk has been confirmed in multiple studies. Obesity-related factors such as estrogen, insulin, and leptin can promote the occurrence and progression of EEC through the IGF-1 signaling pathway and the PI3K/Akt/mTOR pathway (26).

CA125 and HE4 are the most widely studied serum biomarkers in EEC. CA125 is mainly associated with advanced EEC, lymph node metastasis, and myometrial invasion, but has limited sensitivity in early-stage disease. In early EEC, HE4 shows superior sensitivity. According to a study by British scholars (27), the AUC of HE4 for predicting premenopausal endometrial cancer was 0.91, while the AUC of CA125 + HE4 combined detection was 0.77 (95% CI: 0.74–0.81). Although this study was mainly aimed at premenopausal women, its results suggest that HE4 may be a powerful indicator for EEC screening. In addition, Prueksaritanond et al. (28) also demonstrated that preoperative combined CA125 and HE4 levels can improve the diagnostic efficacy of early EC. In this study, CA125 and HE4 were also confirmed as independent risk factors. It is worth noting that we found that even when CA125 and HE4 were still within the normal range, their slight increase may reflect the early progression of EEC, providing a potential early intervention window for high-risk populations.

Low serum albumin has also been shown to be associated with endometrial cancer. In the EC prognosis prediction model established by Chinese scholars (29), low preoperative ALB, high fibrinogen, NLR, and CA125 levels can be used as independent adverse prognostic factors. ALB is a marker of systemic nutritional status and inflammation, which may reflect hypoalbuminemia, cachexia, inflammation, or liver dysfunction (30). In EEC, lower ALB levels are associated with advanced disease and poor survival, which may be because they reflect systemic metabolic stress. This study supports the role of ALB as a predictor, which is consistent with its inclusion in the prognostic models of other cancers. However, its specific intrinsic association with EEC still needs further exploration.

The predictive model developed in this study integrates clinical variables and circulating biomarkers to provide individualized risk estimation for EEC in postmenopausal women undergoing evaluation for endometrial abnormalities. However, this study still has limitations, including the need to verify the robustness of the model in a larger, multi-center population and further explore the temporal association between biomarkers and the development of EEC. Although the AUC of the prediction model in this study was as high as 0.936 (95% CI: 0.9081-0.9631), external validation is still required in an independent cohort to confirm its predictive accuracy and clinical applicability. In addition, future studies should focus on the molecular mechanisms of markers such as PLR and ALB, and combine specific inflammatory cytokines (such as IL-6 and TNF-α) to further clarify their mechanisms of action in EEC.

In the field of diagnosis and treatment of endometrial cancer, future research directions will focus on the improvement of multi-marker combined diagnostic models to improve the accuracy of diagnosis and prognosis. At the same time, artificial intelligence (AI) and imaging omics combined with serum marker data can further optimize the risk stratification and treatment strategies of EEC. In addition, combined with molecular typing, it is expected to promote precision medicine for EEC and provide new ideas for targeted therapy and immunotherapy.

## Data Availability

The raw data supporting the conclusions of this article will be made available by the authors, without undue reservation.
